# Role of interferons in the antiviral battle: from virus-host crosstalk to prophylactic and therapeutic potential in SARS-CoV-2 infection

**DOI:** 10.3389/fimmu.2023.1273604

**Published:** 2024-01-15

**Authors:** Grigore Mihaescu, Mariana Carmen Chifiriuc, Roxana Filip, Coralia Bleotu, Lia Mara Ditu, Marian Constantin, Roxana-Elena Cristian, Raluca Grigore, Serban Vifor Bertesteanu, Gloria Bertesteanu, Corneliu Ovidiu Vrancianu

**Affiliations:** ^1^ Microbiology Immunology Department, Faculty of Biology, University of Bucharest, Bucharest, Romania; ^2^ The Research Institute of the University of Bucharest, Bucharest, Romania; ^3^ Department of Life, Medical and Agricultural Sciences, Biological Sciences Section, Academy of Romanian Scientists, Bucharest, Romania; ^4^ Faculty of Medicine and Biological Sciences, Stefan cel Mare University of Suceava, Suceava, Romania; ^5^ Microbiology Department, Suceava Emergency County Hospital, Suceava, Romania; ^6^ Cellular and Molecular Pathology Department, Stefan S. Nicolau Institute of Virology, Bucharest, Romania; ^7^ Institute of Biology of Romanian Academy, Bucharest, Romania; ^8^ Department of Biochemistry and Molecular Biology, Faculty of Biology, University of Bucharest, Bucharest, Romania; ^9^ ENT Department, University of Medicine and Pharmacy Carol Davila and Coltea Clinical Hospital, Bucharest, Romania; ^10^ DANUBIUS Department, National Institute of Research and Development for Biological Sciences, Bucharest, Romania

**Keywords:** interferons, receptors, signal transduction pathways, SARS-CoV-2, therapeutic role of IFNs

## Abstract

Mammalians sense antigenic messages from infectious agents that penetrate the respiratory and digestive epithelium, as well as signals from damaged host cells through membrane and cytosolic receptors. The transduction of these signals triggers a personalized response, depending on the nature of the stimulus and the host’s genetics, physiological condition, and comorbidities. Interferons (IFNs) are the primary effectors of the innate immune response, and their synthesis is activated in most cells within a few hours after pathogen invasion. IFNs are primarily synthesized in infected cells, but their anti-infective effect is extended to the neighboring cells by autocrine and paracrine action. The emergence of the severe acute respiratory syndrome coronavirus 2 (SARS‐CoV‐2) pandemic in 2019 was a stark reminder of the potential threat posed by newly emerging viruses. This pandemic has also triggered an overwhelming influx of research studies aiming to unveil the mechanisms of protective *versus* pathogenic host immune responses induced by SARS‐CoV‐2. The purpose of this review is to describe the role of IFNs as vital players in the battle against SARS‐CoV-2 infection. We will briefly characterize and classify IFNs, present the inductors of IFN synthesis, their sensors, and signaling pathways, and then discuss the role of IFNs in controlling the evolution of SARS-CoV-2 infection and its clinical outcome. Finally, we will present the perspectives and controversies regarding the prophylactic and therapeutic potential of IFNs in SARS-CoV-2 infection.

## 1 Introduction

Viral infections still cause significant morbidity and mortality globally, despite vaccination, which has majorly contributed to decreasing their burden or even eradicating them. The balance between the viral host conquering strategies and the host immune response dictates the clinical outcome of a viral infection. Therefore, elucidating the intimate mechanisms of the antiviral immune response is essential for developing new preventive, diagnosis, and therapeutic strategies (1).

Viral aggression is initially countered by the innate defense mechanisms located at the level of the epithelial barrier, involving genetic, chemical, and biological factors (2, 3). If the virus surpasses the innate host defenses and starts replicating, the host will respond by triggering an inflammatory response mediated by effector molecules and cells of the innate immune system mobilized from the local blood vessels at the site of infection (4). If the innate immune response fails to clear the infection, even after being augmented by increased production of effector molecules and cells, an adaptive immune response will be initiated (2, 5).

The inflammatory response typically involves four key components: inflammatory inducers, sensors that detect these inducers, inflammatory mediators induced by the sensors, and the target tissues affected by these mediators. The damaged tissue macrophages will recognize, bind, and phagocytose the viral pathogen through their surface receptors and activate an inflammatory response, leading to the accumulation of humoral (plasma proteins such as complement components) and cellular (neutrophils, macrophages, and dendritic cells – DCs) effectors of the innate immunity. Viral, bacterial, and fungal infectious agents show a wide range of associated antigens named “pathogen-associated molecular pattern” (PAMP), quickly recognized by epithelial cells, macrophages, and resident DCs, through innate immunity sensors called “pattern recognition receptors” (PRR) (6). PRR sensors recognize a broad pattern of PAMP antigens and play an essential role in developing innate and adaptative immune responses. The best-known PRR sensors that PAMP activates are classified in 4 families: (i) Toll-like receptors (TLR), (ii) retinoic acid-inducible gene I (RIG-I)-like receptors (RLRs) that includes RIG I, melanoma-associated differentiating protein 5 (MDSA5), and Laboratory of Genetics and Physiology 2 (LGP 2) (iii) NOD-like receptors family (NLR) (NOD- nucleotide-binding oligomerization domain), and (iv) C-type lectin sensors (7, 8). After recognizing the danger signals, the cell activates the molecular effectors mediating the aggression response.

Interferons (IFNs) are essential players in the inflammatory innate response, significantly impacting cellular, tissue, and overall physiological functions (9, 10). IFNs, as suggested by their name, were initially discovered for their remarkable ability to interfere with and counteract viral infections in the 1950s (11). The production of IFNs is triggered by a wide array of pattern recognition receptors (PRRs) (12). Once IFNs are produced, they initiate an intrinsic antiviral state within the cells that detect them, characterized by the activation of interferon-stimulated genes (ISGs), which are instrumental in antiviral defense (13).

The emergence of the severe acute respiratory syndrome coronavirus 2 (SARS‐CoV‐2) pandemic in 2019 was a stark reminder of the potential threat posed by newly emerging viruses. This pandemic has also triggered an overwhelming influx of research studies to understand the different mechanisms involved in the SARS‐CoV‐2 triggered protective or harmful immune responses. Given the critical role of IFNs in controlling viral infections, these cytokines have emerged as vital players in the battle against SARS‐CoV-2 infection (14–27). While IFNs are essential for controlling SARS-CoV-2, they can also potentially contribute to developing severe COVID-19 clinical forms (28–30). This variety and complexity of immune responses is caused by the virus’s ability to evade or manipulate the IFN-mediated host responses, which might not be perfectly tuned to combat this novel pathogen (31).

The purpose of this review is to describe the role of IFNs as vital players in the battle against SARS‐CoV-2 infection. We will briefly characterize and classify IFNs, present the inductors of IFN synthesis, their sensors, and signaling pathways, and then discuss the role of IFNs in controlling the evolution of SARS-CoV-2 infection and its clinical outcome. Finally, we will present the perspectives and controversies regarding the prophylactic and therapeutic potential of IFNs in SARS-CoV-2 infection.

## 2 General characterization and classification of IFNs

IFNs are one of the six groups of cytokines (i.e., interleukins-ILs, tumor necrosis factors-TNFs, chemokines, transforming growth factors-TGFs, hemopoietic growth factors, and IFNs) produced by the host cells primarily in response to viral pathogens. Four types of IFNs, noted I (IFNα with 13 subtypes, IFNβ, IFNϵ, IFNκ, IFNω found in bovines and humans, IFNδ found in pig, IFNτ, IFNς or limitin and trofoblastic IFNτ in ruminants), II (IFN-γ), III (IFN-λ1-4 sharing similarities with the IL-10 family cytokines) and IV were described until now (18, 32–46).

Human IFNs are proteins of 130-170 amino acids with a molecular weight of 20 - 100 kDa, active at temperatures generally lower than 56-60°C and at different pH values (47–51). IFNα and IFNβ (are acid-stable, while IFNγ is acid-labile (13, 52). IFNs exhibit different activities, including the induction of an antiviral state, inhibition of normal and tumor cell multiplication, cell differentiation regulation, and immune function regulation by modulating the expression of major histocompatibility complex (MHC) molecules (48, 53). Type I and III IFNs are the first responders when a viral threat is detected and can be produced by most cell types. In contrast, type II IFNs are primarily generated by specialized immune cells like NK, B, TCD8+, and TCD4+ cells, participating mainly in allergic responses, host defense against intracellular pathogens, and the control of tumor growth. This distinction highlights the diverse roles and functions of different IFNs (54–56). Type IV IFN (IFN-υ or IFN-U), along with its receptors (IFN-υR1 and IL10R2), has been recently discovered in jawed vertebrates (45).

## 3 Inductors of IFN synthesis

In the absence of activating signals, normal cells do not produce detectable levels of IFN. IFN synthesis is triggered by different infective agents: viruses, mycoplasma, rickettsia and chlamydia, ds RNA, synthetic polymers, mannans, and metabolic activators (31, 57–59). The first demonstrated, and most important stimulators of IFN synthesis are the nucleoprotein complexes of ds RNA viruses, as demonstrated by Isaacs and Lindenmann (11). DNA viruses, except Poxviruses, are weak inducers, while the ssRNA genome induces IFN through their ds intermediates produced during replication (60). Inactivated viruses and those infecting non-permissive substrates are also IFN synthesis inducers (61). In mononuclear leukocytes, IFN synthesis is induced by the viral envelope glycoproteins (62).

The main classes of PAMP receptors are i) TLR sensors harboring a cytoplasmic domain with a sequence similar to Toll/IL1R (TIR) receptor and stimulating IFN I, IFN III, and other mediators involved in inflammatory and adaptative immune responses synthesis (37, 63–66); ii) NLR (NOD-like receptor) receptors are cytosolic proteins that control this compartment for intracellular aggressor signals; iii) RLR sensors (retinoic acid-inducible gene I) are a vast cytosolic helicase protein family acting as the most important sensors for ds RNA, ss RNA, and DNA viruses (67–71); iii) cGAS (Cyclic GMP-AMP synthase), secondary endogenous messenger sensing the cytosolic DNA and consequently synthesizing the second messenger, cGAMP, further binding and activating STING (stimulator of IFN genes), which on its turn, activates TBK1 kinase that induces the STING-dependent IFNb synthesis through the IRF 3 (IFN regulatory factor 3) (72–74); iv) Type C lectin sensors (e.g., mannose receptors) are carbohydrate structures associated with macrophage, CD, and Langerhans cells membrane that recognize carbohydrate surface structures of bacteria, viruses, and parasites (7, 75); v) ALR sensor (Absent In Melanoma 2 = AIM-2-Like Receptor), detected in odontoblast layer (76), recognizes cytosolic and pathogen DNA and is inductive of IFN I synthesis (77, 78).

ISG can be activated by the common mechanisms of the other PRR sensors: activator phosphorylation and nuclear translocation of cytoplasmic factors IRF3, IRF7, and NF-kB (79).

Activation of IFN synthesis is a complex process consisting of the following: (i) viral antigen recognition mediated by macrophages, DCs, and Langerhans epithelial cells receptors, (ii) signal transduction through the adaptor kinase pathway, (iii) phosphorylation and nuclear translocation of transcription factors IRF3/IRF7 (IFN regulatory factor), and (iv) stimulation of ISG transcription (80–83). IRF and NF-kB activation trigger two antiviral programs: (i) cell defense by induction of antiviral status mediated by IFN I, which in its turn activates leukocytes, especially neutrophils and IFN III, and (ii) activation of an extended ISG genes series (48).

## 4 IFNs receptors and signal transduction pathways

The IFN molecule has two domains with relatively constant amino acid sequences: the N-terminal domain that forms the B (binding) situs on the cell receptor, and the other A (activity) in the C-terminal domain that seems to modulate receptor binding and mediate the adaptative and innate immune responses (50). Their main role is the induction of viral multiplication inhibitor protein synthesis. IFN receptor distribution is strictly regulated, dictating the cell capacity to answer IFNs.

**Figure 1:**
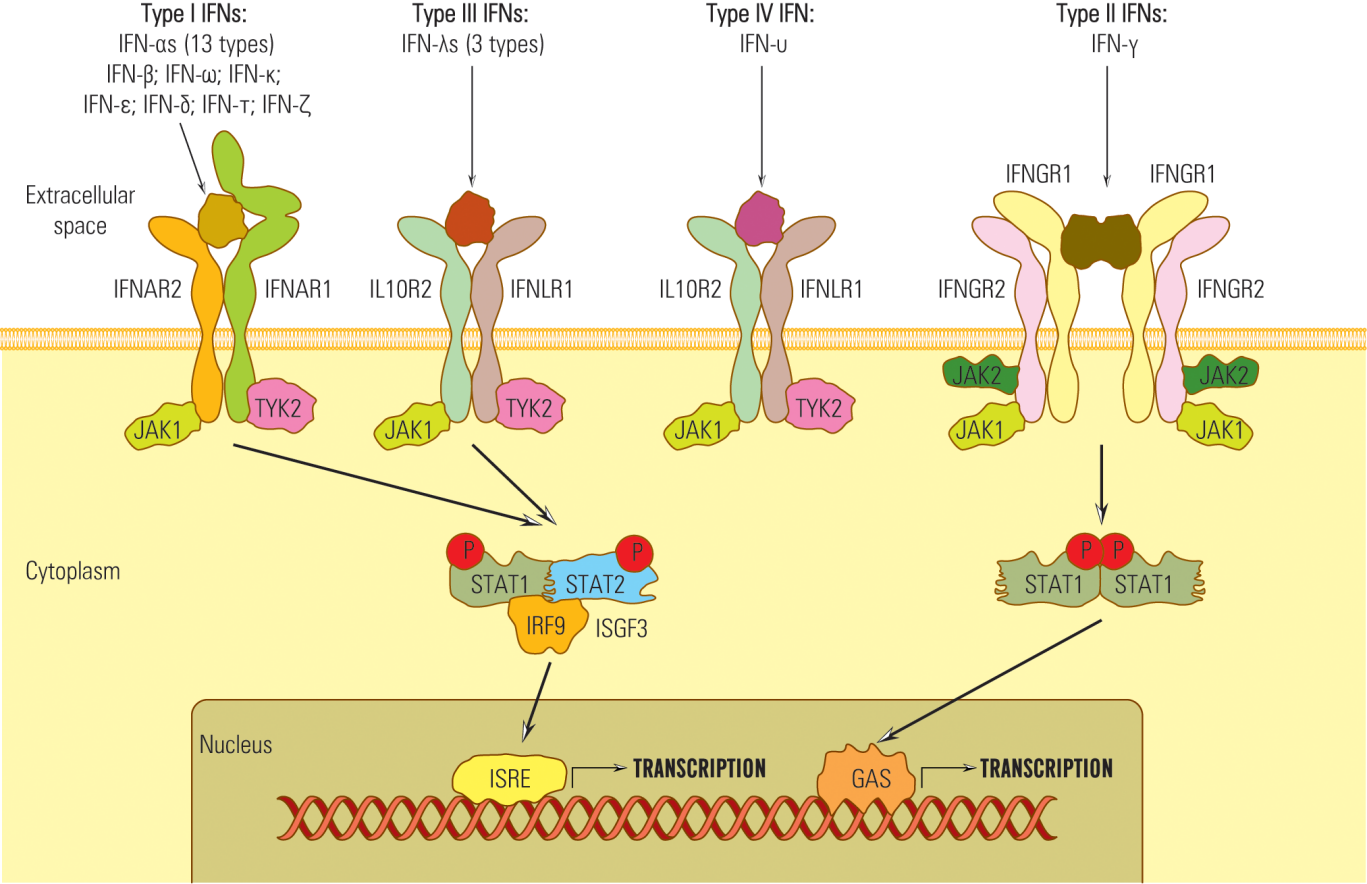
Schematic representation of the IFN receptors network.

Despite their diverse amino-acid homologies, all type I IFNs transmit signals through a common heterodimeric receptor consisting of low-affinity (IFNAR1) and high-affinity (IFNAR2) receptor components (84) (**Figure 1**).

In order to transmit signals via the JAK/STAT pathway, both IFNAR1 and IFNAR2 receptors form associations with tyrosine kinase 2 (Tyk2) (in the case of IFNAR1) and Janus kinase 1 (JAK1) in the case of IFNAR2 (85, 86) (**Figure 1**). These kinase associations are essential for the phosphorylation of the receptor and the recruitment of STAT (signal transducer and activator of transcription) proteins to the receptor complex (87, 88).

In contrast to type I and type II IFNs, which have distinct receptors, the IFNλ share a common receptor, IFNLR1, with another group of cytokines, including IL-10, IL-22, and IL-26 (IL10RB) (34). All type III IFNs transmit signals through a common heterodimeric receptor consisting of two subunits: IFNLR1, also referred to as IL28Rα, and IL10Rβ (84, 89–91). The signaling complex employed by type III IFNs comprises four transmembrane-spanning receptors, which include two copies of each of the high-affinity receptor IFNGR1 and low-affinity receptor IFNGR2 (84) (**Figure 1**). The activation of the JAK/STAT signaling pathway in response to IFNλ binding to its receptor complex is facilitated by JAK1, associated with IFNLR1, and respectively by Tyk2 linked with IL10RB (39).

**Figure 2:**
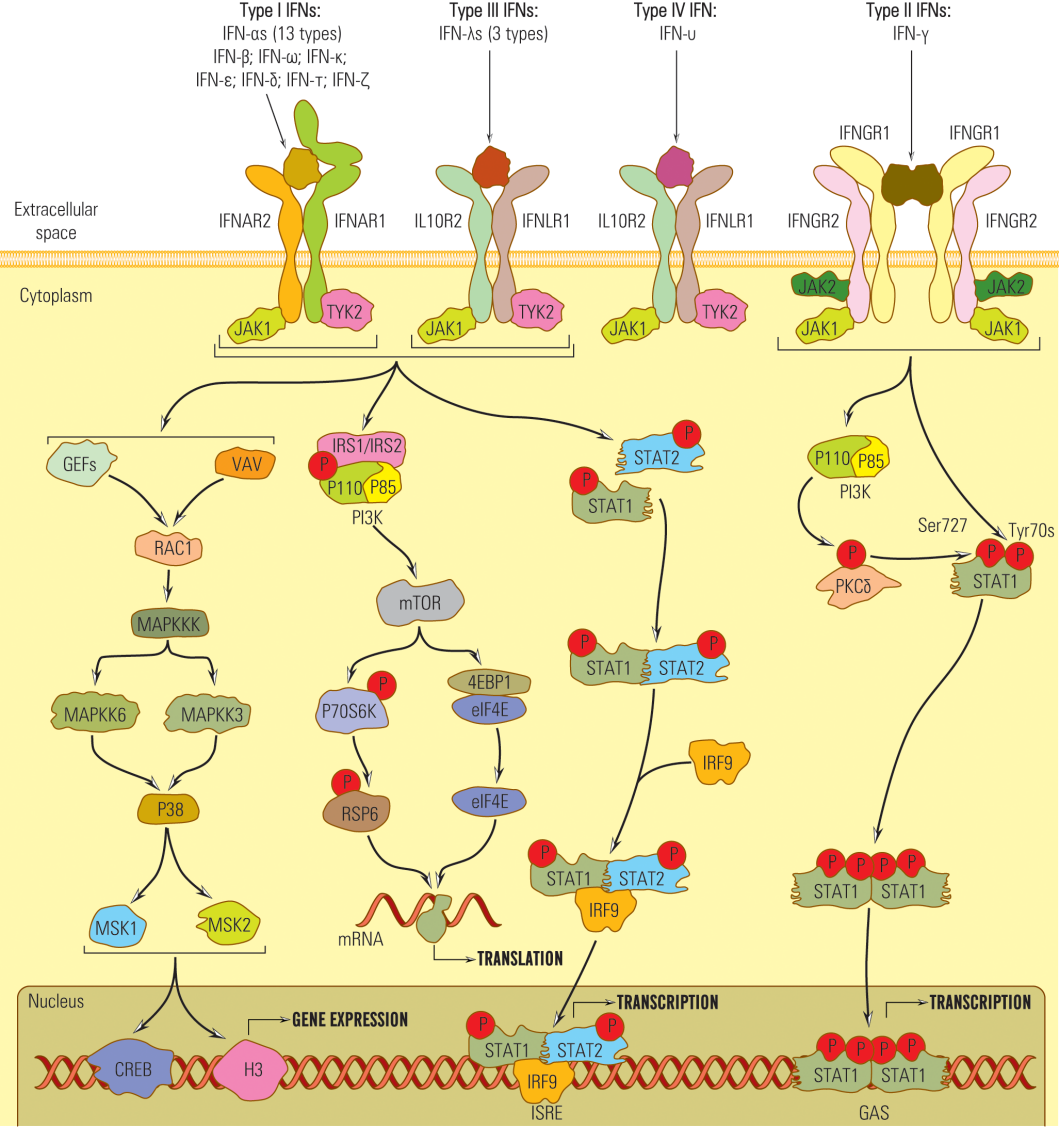
Schematic representation of the IFN signaling pathways.

Therefore, IFNs I, II, and III signals are essentially transduced by JAK/STAT pathway, but other pathways might be involved, which explains the diversity of IFNs biological effects (50, 92, 93) (**Figure 2**).

Notably, type IV IFN shares a C-terminal sequence with type I IFN and utilizes the same signal-transduction receptor, IL10R2, as type III IFN. This suggests a common ancestral origin for type IV IFN and type I/III IFNs (45, 94).

Upon activation, STAT kinases attract IRF9 and form the heterotrimer complex STAT1-STAT 2-IRF-9 known as ISGF 3 (IFN stimulated gene factor 3) (95). ISGF 3 complex migrates into the nucleus, activates ISRE (IFN stimulated response element) belonging to the ISG gene promoter, and starts transcription (96).

The STAT combinations that bind ISRE are STAT 1-STAT 2, STAT 2-STAT 3-6, or STAT 2 homodimers that generate diversity among IFN-activated genes and effects, e.g., STAT 1 recruitment induces pro-inflammatory, antiproliferative, and proapoptotic effects; STAT 3,4,5 stimulate T lymphocyte proliferation (48, 56, 97, 98).

IFN III activates the STAT 1-STAT 2 pathway and regulates gene transcription through ISRE or by GAS elements (56, 89, 99) (**Figure 1**). STAT complexes that do not attract IRF 9 bind to the GAS site (IFN-γ activated site) and activate ISG promoters. For example, IFN-γ does not induce ISGF3 complex formation and, consequently, does not activate genes with only ISRE in their promoters (53, 100).

Besides the JAK/STAT pathway, IFN I, II, and III, by JAK kinases, can activate other non-STAT pathways: MAPK (mitogen-activated protein kinase), crucial in the activation of antiproliferative and antiviral genes depending on IFNI and PI3K pathway (35, 101).

Some genes can be induced in the absence of IFN. Some are activated by IRF1, IRF3, IRF 7, NF-kB, or IL-1 (56, 102).

## 5 Antiviral IFN activity

Even though IFNs are not effector molecules, they induce an antiviral state that depends on extracellular IFN level, cell type, viral virulence, functional status of immune effectors, and activity of the molecular complex of PRR sensors, signal transduction pathways, and IFN codifying genes.

The *in vitro* IFN synthesis is activated in the first 2-3 hours and reaches a maximum in 16 hours after macrophage infection by the influenza virus (103). Type I IFN, produced by the infected cells simultaneously with the viral multiplication cell cycle, is released from cells immediately after synthesis, and diffuses toward the non-infected cells.

IFN I and III mediated antiviral state is a consequence of two mechanisms (104). First, in most virus-cell systems, viral multiplication inhibition results from interference with mRNA translation: IFN inhibits the binding of ribosomes to viral messengers. Secondly, in infected cells, IFN activates the expression of hundreds of ISG cluster genes that mediate pleiotropic effects, counting antiviral, antiangiogenic, immunomodulatory, cell cycle inhibitory, apoptotic, and antitumor activities (105–108). Genes activated by IFN I and IFN III codify a variety of antiviral effectors (109–111). ISG activation leads to mRNA synthesis, but some hundreds of ISG encode non-coding RNA. Other molecules, besides IFNs, could directly activate some ISGs, such as IRF, NF-kB, or IL-1 (112). Others have a basic transcription level, the IFNs only exhibiting a stimulatory or repressor effect. The response to different viruses is individual (109).

The antiviral protective effect induced by IFN is present in all stages of the multiplication cycle: cell entry, decapsidation, transcription, translation and replication of viral RNA, assembly, and release of virions. Infections with IFN-sensitive viruses are more severe in laboratory animals previously injected with anti-IFN I or actinomycin D (a specific protein synthesis inhibitor) (113, 114).

The most essential antiviral effectors with IFN I induced synthesis are RNA-dependent protein kinase (PKR) and 2’ 5’ oligo-adenosine synthetase (OAS), specific adenosine deaminase for RNA (ADAR), the product of gene 56 GTP ase, Mx, etc. (54, 97, 115). The antiviral effect was proved especially for PKR and for 2’-5’-OAS/RNase L system. Both are constitutively expressed in normal cells as inactive latent forms. The minimum mRNA level for these cells is stimulated by IFN I (109, 116–118). PKR is one of the four kinases from mammalian cells, the most critical antiproliferative and antiviral effector induced by IFN I (119). The enzyme is mainly found in the cytoplasm (80%) associated with ribosomes and is more phosphorylated than the nuclear one. In the absence of inductors, PKR is synthesized in a lower amount, non-phosphorylated, monomeric, and inactive. After binding of IFN I to membrane receptor or/and in the presence of dsRNA or influenza nucleoprotein, PKR level increases, dimerizes, self-phosphorylates, and phosphorylates the eukaryotic protein synthesis factor (eIF2α) and inhibits viral mRNA translation (67, 120, 121). The 2’-5’oligoadenylat synthetases (OAS) bind dsDNA and are activated by conformational change. The major role of 2’-5’OA is the activation of RNase L, constitutively synthesized and detectable in all animal cells, that, in its turn, destroys viral RNA and blocks viral multiplication, and also induces apoptosis (6, 122–124). The 2’-5’OAS enzyme could exhibit antiviral activity also by RNase L independent pathways.

Other antiviral effectors are: i) protein 56 (p56) that binds the 3e initiating factor of the eukaryotic cell (eIF3e), inhibits mRNA translation initiation, and thus activates apoptosis (125, 126); ii) DICER protein from the dsRNA binding family (DRBP- =ds binding protein) is involved in gene inactivation mediated by RNA interference (67).

IFN a/b has an indirect protective effect by decreasing the permeability of the digestive and respiratory tract mucosa and consolidating the blood-brain barrier (116). It has been shown that neurons, astrocytes, and microglia synthesize IFN I with an anti-pathogenic protective effect (127, 128). Also, IFN stimulates the major histocompatibility complex molecules (MHC) from class I and II and their co-stimulators, which are encoded by ISG genes, on the surface of antigen-presenting cells (APC) (100). IFN I also stimulate chemokine-mediated APC migration (48).

IFN I protects cells from the viral cytopathic effect but does not completely eliminate the infection. Antiviral protective status IFN lasts several days and can be reinduced (127).

Inadequate IFN I synthesis amplifies the pathogenic effects of acute and persistent viral infection by immunosuppressive effects and aberrant inflammatory reactions (129). IFN I chronic synthesis can be associated with clinical manifestations: infectious diseases, neoplasia, chronic inflammation, and autoimmune diseases. Mutations in genes codifying synthesis for IFN, IFN receptors, or activator signal transduction pathways might generate a risk of developing viral or bacterial infections (130). Some ISG genes, such as adenosine deaminase (ADAR), can stimulate certain viruses’ multiplication (131, 132).

IFN γ is a pleiotropic cytokine with contradictory effects reported in viral infections, depending on the virus, the intensity of the innate and adaptative immune response, pro-inflammatory cytokine synthesis rate, and underlying pathology. It stimulates cellular immune response, protects against *Mycobacterium tuberculosis*, inhibits the multiplication of certain viruses, activates macrophages and reactive oxygen species (ROS) release, and induces macrophage polarization to M1 pro-inflammatory phenotype but maintains the M1/M2 ratio (55, 133). M1 macrophages have microbicidal activity and release pro-inflammatory cytokines (IL-1β, IL12, TNFα) and M2 IL-10 (anti-inflammatory) and TGF (tumor growth factor) (134). On the other hand, IFN-γ stimulates immunotolerance in chronic viral infection, induces pro-inflammatory cytokine synthesis in SARS-CoV-2 infection (cytokine storm), determines lesions of the lung epithelium, microvascular endothelium, ischemia, pulmonary fibrosis (18). Other authors reported the blocking of experimental liver fibrosis by IFN-γ (135).

Type III IFN regulates the activity for several gene sets and determines biological activities similar to IFN I, the only difference being that IFN III produces limited pro-inflammatory effects (136).

The recent discovery of the type IV IFN system and its antiviral functions still raises several important questions and needs future research to elucidate signaling networks and if the regulatory relationships of type IV IFNs with the other IFN types are synergistic, antagonistic, or independent. Secondly, while type IV IFN has demonstrated strong antiviral properties, it’s important to explore whether it also exerts regulatory functions against other types of pathogens, such as bacteria, fungi, parasites, etc., similar to the type I and II IFNs (94). As a cytokine, it’s important to explore the broader regulatory functions of type IV IFN in various immune cells, including lymphocytes, macrophages, DCs, thrombocytes, neutrophils, etc., and its impact on inflammation, phagocytosis, or other immune responses.

## 6 IFNs in SARS-CoV-2 infection

More than half of human infections are zoonotic, and of the 224 human infective viruses, 88% are zoonotic (137). However, only a few adapted to a human host and initiated pandemics in the last 200 years: H1N1 influenza virus (1918) from birds (138), swine variants of influenza A (2009 pandemics –pdm-09) (139), avian influenza A H7N9 (140); HIV from *Macaccus* (32.7 million deaths) (141); hepatitis C virus (unknown origin, infected more than 70 million people) (142); MERS from camels (143); SARS-CoV-2 associated with *Civeta civetictis*, bats and pangolin (144); Hendra and Nipah viruses (paramyxoviruses) spread by bats (145); encephalitis agents isolated from horse and pig (146). Other viruses with animal-to-human transmission belong to rodents: Hantavirus and Machupo arenaviruses, Lassa, and Junin. In most zoonotic infections, humans represent the final host (147).

IFN I is essential to eliminate the virus and curtail the immune response swiftly (90), being vital in regulating T effector cells, responsible for virus elimination, and for the differentiation of regulatory CD4+ T cells that produce the inhibitory cytokines like IL-10. Blocking IFN signaling during MERS-CoV infection in mice reduced the development of virus-specific CD4+ and CD8+ T cells (148). Studies on IFN I receptor knockout (IFNAR-/-) mice have shown that this can lead to increased pathology due to elevated production of proinflammatory cytokines triggered by the viral infection (149). Response of the target cells to specific cytokines, including IFN I, is regulated by IFN concentration, receptor expression, and viral mechanisms for counteracting the immune response. Chronic IFN I synthesis could have pathologic effects, such as stimulation of antigen presentation and activation of more lymphocyte clones, including self-reactive ones, that can initiate an autoimmune response. The reverse IFN effect synthesizes IL-10, a cytokine with immunosuppressive and pro-apoptotic effects (102).

Experimental data shows that respiratory epithelial cell (nose, oropharynx, nasopharynx, larynx, sinuses, conjunctiva) synthesis of IFN I seems to play a determinant role in the dynamic and severity of SARS-CoV-2 infection (150). The rate of transcription for IFN, AR1, JAK1, and TYK2, transducers of IFN synthesis activating signal (ISG), is higher in patients with mild or moderate infection associated with the increased plasma level of IFN, by contrast with a decrease in ISG expression mediated by inhibitor genes MX1, IFITM1, IFIT2 and decreased IFN I blood level in patients with severe infection (151, 152). Infection severity is associated with family deficiency of ISG or IFN I encoding genes and anti-IFN I autoantibodies (28, 153). Macrophages, DC, and keratinocytes produce IFN κ, and its protective antiviral functions are inhibited in SARS-CoV-1 infection (48). DC produces IFN ω and exhibits antiviral activity against SARS-CoV-2, specific antibodies detected in COVID-19 patients with severe pneumonia (153, 154).

However, while early IFN I production is crucial for an effective T cell response (reducing the SARS-CoV-2 density, severity, and duration of clinical infection), delayed IFN response can inhibit T cell proliferation and result in T cell exhaustion and death (155, 156). Thus, timing of IFN synthesis during infection is essential for stopping infection evolution.

Also, a high blood level of IFN I is not always a marker for viral protection. Longitudinal analysis of viral load for respiratory tract epithelium in critically ill patients with COVID is proportional with IFN-α, IFN-γ, and RNF level, proving that SARS-CoV-2 multiplication is not always regulated by IFN I (28, 157, 158).

The presence of lung injury in severe COVID-19 cases suggests a potential failure to activate immunosuppressive mechanisms promptly. Patients with more severe COVID-19 symptoms tend to have lower counts of regulatory T (Treg) cells influenced by IFNs (159). The decreasing number of Treg cells raises the hypothesis that dysregulated IFN responses elicited by SARS-CoV-2 may impact Treg cell generation during the recovery phase of COVID-19. Future studies should investigate the role of IFN dysregulation in shaping T cell responses and how it may, in turn, affect antibody responses since CD4+ T cell activation is crucial for B cell immunity. A deeper understanding of these interactions will provide valuable insights into the immune response during COVID-19 (160).

Existing comorbidities decreased the immune response and increased the pulmonary pathological process mediated by pro-inflammatory IL produced by macrophages. Pre-stimulated macrophages by external inflammatory factors had the same effect (28, 161, 162).

Non-adequate quantitative response or delayed IFN synthesis in SARS-CoV-2 infection induced the activation of many ISG genes with immunopathologic potential, such as overexpression of genes encoding pro-inflammatory cytokine synthesis, exacerbating the inflammatory reaction (163). In the plasma of critically ill COVID-19 patients, IFN I, IL 6, and TNF α had high concentrations, showing increased IFN I activity mainly from pDCs and neutrophils, that generate a cytokine storm (164, 165).

Regarding type II IFN, the results of the cross-sectional analysis conducted by Piater and collaborators in 142 infected patients show that in most COVID-19 patients, IFN-γ-mediated biochemical pathways were still strongly activated after 60 days. The authors observed that the ongoing activation of IFN-γ-mediated pathways might influence the further course of reconvalescence, and the continuous immune activation might go along with enhanced demand for nutrients like amino acids and vitamins (24). Mansoor and collaborators aimed to investigate the crosstalk between host immune response mediated by cytokines and the severity of SARS-CoV-2 infection by assessing cytokine expression in 136 infected patients. In this regard, the authors measured the expression levels of 12 genes encoding inflammatory, anti-inflammatory, and regulatory cytokines using QRT-PCR in hospitalized patients with severe infection and found that IFN-γ could be a potent marker of disease severity (22). Primorac and colleagues designed a study involving 303 participants who were tested for the analysis of IFN-γ concentration and the detection of human antibodies of the immunoglobulin class IgG against the S1 domain of the SARS-CoV-2 spike protein. The statistical analysis revealed a significant difference in the IFN-γ concentration between participants who had experienced reinfections and those who had not been infected. Participants who had not been infected or reinfected with SARS-CoV-2 after vaccination and before SARS-CoV-2 infection displayed a notably higher level of cellular immunity. Additionally, among individuals who had not received additional vaccination, those who had experienced infection or reinfection had significantly lower IFN-γ levels than uninfected participants. These findings suggest that cellular immunity, as measured by IFN-γ concentrations, has a lasting impact and is crucial in preventing infections and reinfections, especially in the context of emerging SARS-CoV-2 variants of concern (25).

Suzuki and collaborators conducted a study to investigate the potential of 71 humoral factors as predictive markers of COVID‐19 in 188 patients diagnosed with COVID‐19 using antigen or nucleic acid amplification tests. The authors showed that IFNλ3 predicted subsequent oxygen demand better than other humoral factors (e.g., CRP, LDH, lymphocyte fraction) in patients in the early phase of COVID‐19 without supplemental oxygen demand. IFNλ3 may effectively predict whether a patient with COVID‐19 will require medical intervention, such as oxygen supplementation, at an earlier point before the patient presents with respiratory failure. In conclusion, in patients with COVID-19 who do not require supplemental oxygen for the first week after the onset of the disease, the serum IFNλ3 level is a highly accurate predictor for the likelihood of needing oxygen support later (21). These findings hold significant importance in making early decisions regarding patient placement and initiating timely therapeutic interventions. Looking ahead, IFNλ3 could potentially serve as a valuable tool for enhancing the prognosis of COVID-19 patients while alleviating the strain on healthcare facilities.

A recent study conducted by Matic and collaborators determined that the presence of the most frequent functional single nucleotide polymorphisms (SNPs) of the two most important IFN-λs coding genes, namely IFNL3 and IFNL4, could alter the likelihood of SARS-CoV-2-infected patients to develop a more severe form of the disease. This clinical study involving 178 COVID-19 patients revealed that carriers of IFNL3 and IFNL4 minor alleles are less likely to progress from mild to moderate COVID-19, that is, to develop COVID-19-related pneumonia. Also, the authors observed that the likelihood of pneumonia development remained significantly associated with IFNL4 polymorphism, especially in females. These results suggest that IFNL4 rs12979860 and rs368234815 polymorphisms could predict the risk of COVID-19-related pneumonia development in females (23).

## 7 Viral strategies against IFNs

During the SARS-CoV-2 pandemic, the interest in understanding the mechanisms by which animals tolerate viral infections has increased. Rodents and bats have developed strategies for overcoming or deleting the immune response (166). Bats have an increased response to viral RNA and a decreased response to cytoplasmic DNA viruses because of the lack of genes codifying DNA cytoplasmic sensors. In humans, most viruses induce synthesis of IFN and ISG gene transcription stimulating factors, but the most virulent neutralize the IFN response. For most viruses, the gene expression inductor for IFN α/β is dsRNA. Many viruses generated strategies that prevent dsRNA exposure in the cytoplasm (167–169). In reoviruses, dsRNA remains inside the capsid during the whole viral cycle. Also, most replication intermediates are associated with viral proteins covering ds viral RNA regions; thus, only a small amount of dsRNA is exposed (170, 171).

In most cases, viruses inhibit the innate immune response by the action of nonstructural and structural proteins that inhibit transmission of IFN synthesis activating signals (172, 173). Most viruses encode molecules that destroy PKR, inhibit the interaction between PKR and ds RNA, and inhibit PKR phosphorylation (173–175). Poxviruses encode proteins that inhibit the host immune response by blocking IFN, TNF, IL-1 chemokines synthesis, and transduction of apoptosis activating signal (176). One of the mechanisms by which bats tolerate the SARS-CoV-2 infection is that ISG transcription is constitutively expressed at low levels, and regulatory genes are lacking. Inflammation is limited by inhibiting TNF-α expression associated with a decreased level of NLRP3 inflammasome activation. Bats response to viral infection is, in fact, anti-inflammatory, being associated with the expression of IL-10 (177, 178).

**Figure 3:**
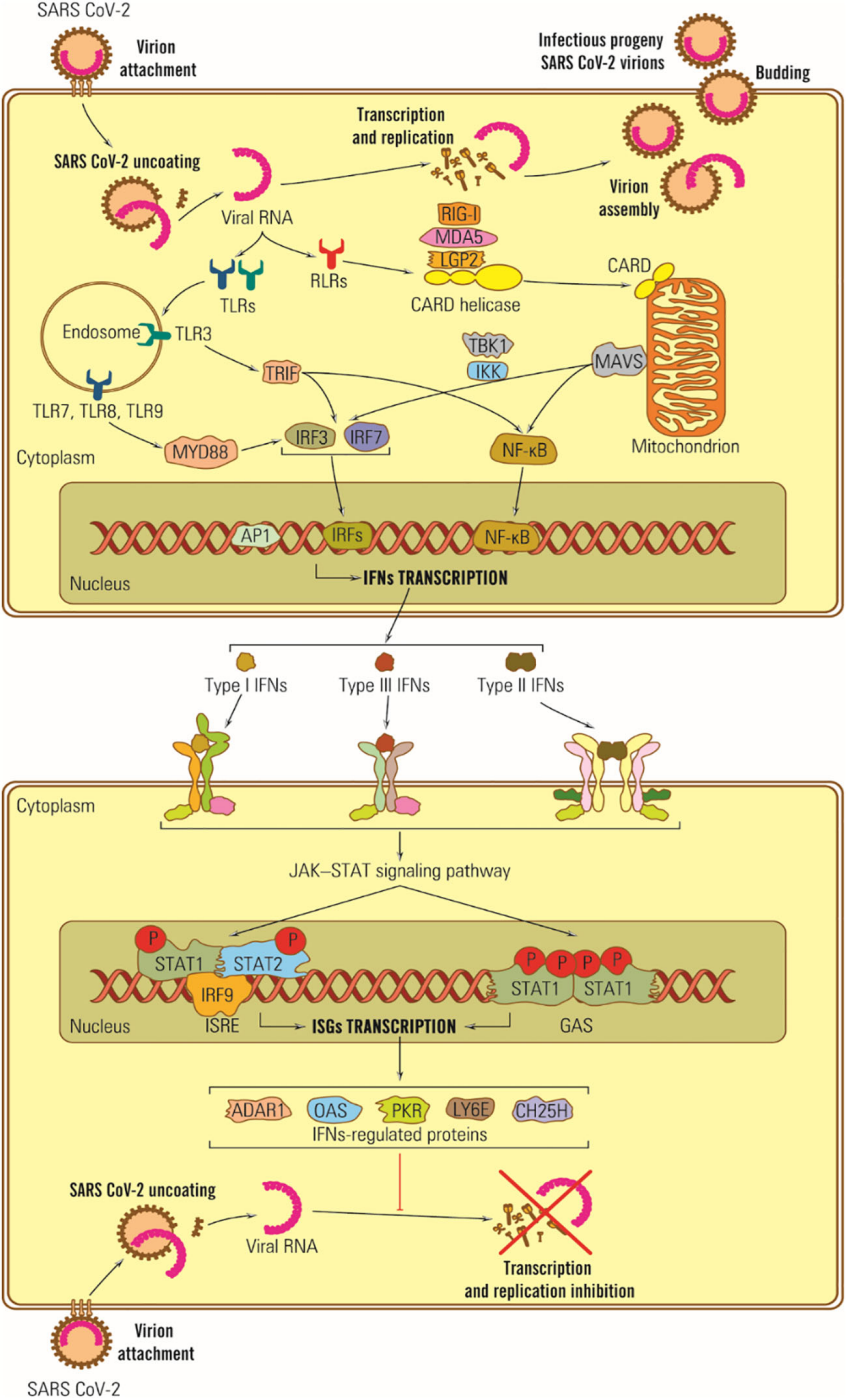
Schematic representation of IFN antiviral effects in SARS-CoV-2 infection. Viral RNA is recognized by TLR-3, -7 and -8 located on the endosomal membrane, and protein E is directly bound to TLR-2. Its dysfunction plays a vital role in the CNS response to SARS-CoV-2 infection, potentially triggering neurodegenerative lesions. Ds subgenomic RNA synthesized in mRNA transcription is recognized by RIG I and MDA-5 (185).

In the case of SARS-CoV-2, some structural proteins and most SARS-CoV-2 nonstructural proteins (nsp) are involved in counteracting the IFN synthesis activating signals by different pathways: (i) M protein can interact with the MDA5 sensor, or with MAVS adaptor protein or TBK1, part of the IFN I synthesis activating signal transduction chain, (ii) M protein induces ubiquitin and TBK1 degradation and blocks transduction of IFN synthesis activating signal (179), (iii) nsp1 inhibits cell mRNA translation and IFN synthesis (180, 181), (iv) ORF 9b protein inhibits the signaling pathway for RIG –I/MDA5-MAVS adaptor proteins TRIF and STING for TLR-3 TRIF signaling pathway as well as cGAS-ATING pathway, the sensor of cytosolic DNA by which IRF 3 phosphorylation and nuclear translocation are blocked (182), (v) the SARS-CoV-2 ARN-polymerase blocks IRF 3 nuclear translocation as well as ORF 6 protein (183), (vi) ns 3 and ns 5 proteases cleave viral polyproteins and also cell proteins, blocking the IFN receptors by direct action, (vii) N protein covers the genomic RNA, inhibits dsRNA recognition by RIG I, and inhibits phosphorylation of STAT 1, and STAT 2 kinases and their translocation in the nucleus (98, 184), (viii) nsp 6 and nsp 13 bind TBK1 and delete the IRF activation; ix) other ns proteins block the activator signal for STAT 1/STAT 2 phosphorylation and their translocation in the nucleus (98, 183), and (x) coronaviruses replicate in structures limited by membranes that protect viral RNA from cell sensors (**Figure 3**).

The ability of SARS-CoV-2 proteins to interfere with IFN I questions the use of the whole virus as a vaccine. On the other hand, vaccinating with specific SARS-CoV-2 proteins that lack IFN I regulatory activity, like the S-protein, may lead to enhanced IFN I production and a stronger immune response (186).

## 8 Potential of IFNs for the therapeutic management of COVID-19

The dysregulation of IFNs in COVID-19 highlights its importance in the disease’s development and its potential as a therapeutic target. IFNs administered prophylactically can create an antiviral state in cells, potentially preventing early-stage viral infections (151). In animal studies, early IFN treatment before viral replication peak was beneficial, while late administration hindered viral clearance and worsened immunopathology (155).

An experimental trial in 2020 demonstrated that daily IFN-α nasal drops, along with standard protective equipment, protected at-risk healthcare workers from COVID-19 for 28 days without adverse effects (NCT04320238).

Clinical evidence suggests that administering IFN I interferon early in SARS-CoV-2 infection is more effective in reducing disease severity and mortality compared to late administration (14, 20). The early treatment can probably speed up viral clearance, leading to quicker recovery and a decreased risk of severe illness. Moreover, early-stage IFN treatment may also play a role in reducing virus transmission (187).

Pandit and collaborators led a clinical trial to investigate the effectiveness and safety of pegylated interferon alfa-2b (PEG IFN-α2b) in 40 patients with moderate COVID-19. The primary endpoint was the improvement in clinical status on day 15, measured by the WHO 7-point ordinal scale. Overall, 19 subjects who received PEG IFN-α2b had improved clinically on day 15, and 80% had a negative RT-PCR result on day 7. Mild adverse events were reported for eleven subjects. This study highlights the significant improvement in clinical status on day 15 due to faster viral reduction due to the PEG IFN-α2b administration in moderate COVID-19 subjects (17). Levy and collaborators reported the safety of a single subcutaneous injection of Peg-IFN-α2a in two patients with inborn errors of the TLR-3 and IRF-7, affecting the production of type I IFNs and predisposing to severe COVID-19. Both patients reported a rapid decrease in the symptoms and signs present at admission following the administration of Peg-IFN-α2a, which suggests that these types of patients may benefit from the very early administration of type I IFN. Furthermore, this observation suggests that the earliest possible administration should be considered in trials of type I IFN-based regimens for treating SARS-CoV-2-infected patients at risk of developing severe disease (16).

Recently, Jhuti and collaborators reviewed eleven studies reporting the benefits of IFN therapy for the treatment of SARS-CoV-2 and, based on the findings, formulated several recommendations to be taken into account in IFN therapy: (i) standardized outcome measures, (ii) dedicated analyses by interferon type, (iii) analyses of effectiveness by disease stage, (iv) exploration of higher IFN dosage, and (v) cost analysis (19).

Although clinical studies combining IFNs with antivirals for SARS-CoV and MERS-CoV yielded inconclusive results due to variable administration timings and comorbidities (15), however, the most effective approach seems to be combining interferon with other repurposed antiretroviral drugs, such as lopinavir, ritonavir, and remdesivir (188, 189).

A recent finding has shown that that ACE2 is an IFN-stimulated gene in the human airway epithelial cells. This finding raises questions about whether prophylactic or therapeutic IFN administration might enhance SARS-CoV-2 entry and replication during disease progression (15).

Recent studies found that patients with COVID-19 exhibited an increase in the production of IFN-γ, even before the release of antibodies. As a result, this potent cytokine appears to be the primary mediator of innate and adaptive immune responses during SARS-CoV-2 infection and a potential candidate for therapeutical approaches (18, 190).

Myasnikov and collaborators conducted a randomized study including patients with moderate COVID-19 infection, aiming to assess the effect of subcutaneous administration of IFN-γ (500,000 IU, s/c, daily, once a day, for five days) in patients with viral pneumonia on the changes in vital signs and the duration of hospital stay. The study showed that IFN-y resulted in more favorable changes in the stabilization of vital signs, as well as in reduced length of fever and hospital stay by two days, which suggests a positive effect of this substance on the recovery processes in patients with moderate COVID-19. Notably, patients who received recombinant IFN-γ experienced no progression of respiratory failure and required no transfer to the intensive care unit. These results confirm the positive effect of IFN-y on the clinical stabilization and recovery rate of patients with community-acquired pneumonia and viral infections (191). In a study led by van Laarhoven and collaborators, five critically ill COVID-19 patients with severe defects in cellular immune responses, high SARS-CoV-2 viral RNA loads, and no respiratory improvement were treated with IFN-γ (100 μg subcutaneously, thrice weekly). Bronchial secretion was collected every 48 hours for routine diagnostic SARS-CoV-2 RT-PCR and viral culture. A rapid decline followed IFN-γ administration in SARS-CoV-2 load and positive-to-negative viral culture conversion. The results revealed no signs of hyperinflammation, allowing us to consider IFN-γ as adjuvant immunotherapy in a subset of immunocompromised COVID-19 patients (192).

Despite these promising results, in a clinical trial conducted by Roquilly and collaborators, the results indicated that interferon gamma-1b treatment (100 µg interferon gamma-1b every 48 hours for a period of 1 to 9 days) did not significantly reduce the incidence of disease or death in the first 28 days. In addition, the study was stopped due to safety concerns about interferon gamma-1b treatment (193). These divergent outcomes suggest that further research is needed to determine the efficacy of IFN-γ in both prophylaxis and treatment of COVID-19.

Research on the antiviral properties of interferon lambda (IFN-λ) in the context of intestinal infections has emphasized its enduring and non-inflammatory characteristics. As a result, numerous studies have delved into the prospect of utilizing IFN-λ in COVID-19.

In a study conducted by Feld and collaborators, the benefits of administering a single subcutaneous injection of pegylated IFN-λ were discussed, particularly in the treatment of mild to moderate COVID-19 within the first seven days of symptom onset or upon the first positive swab in asymptomatic cases (194). However, in another randomized, placebo-controlled study involving 120 patients with mild to moderate COVID-19, the subcutaneous administration of pegylated IFN-λ1 within 72 hours of diagnosis did not lead to a shorter duration of SARS-CoV-2 viral shedding nor did it result in symptom improvement (195). These divergent outcomes suggest that further research is needed to determine the efficacy of pegylated IFN-λ in both prophylaxis and treatment of COVID-19 due to the uncertainty surrounding its effects. Also, the exact timing for applying IFN-λ-based therapeutics could be crucial: it should be earlier to significantly reduce the viral load and thus decrease the overall severity of the disease.

More recently, Reis and collaborators conducted a randomized clinical trial (NCT04727424) aiming to investigate the efficacy of a single dose of pegylated IFN-λ in vaccinated adults with SARS-CoV-2 infection from Brazil and Canada. In total, 933 patients received pegylated IFN-λ(single subcutaneous injection, 180 μg), and 1018 received placebo (single injection or oral). This study revealed that the vaccine’s effectiveness remained consistent across different dominant virus variants, irrespective of the patient’s vaccination status. Among individuals with a high initial viral load, those who were administered pegylated IFN-λ showed a notable reduction in viral load by the seventh day compared to those who received a placebo. Furthermore, the incidence of adverse events was comparable in both groups, indicating the treatment’s safety. The authors concluded that among predominantly vaccinated outpatients with COVID-19, those who received a single dose of pegylated IFN-λ had a significantly lower likelihood of being hospitalized or visiting the emergency department than those who received a placebo (26).In addition, Santer and colleagues aimed to determine if a peripheral immune cell response to therapeutic administration of pegylated IFN-λ *in vivo* could be detected. In this context, the authors performed single-cell RNA sequencing (scRNAseq) on 9 patients receiving pegylated IFN-λ. ScRNAseq was performed to investigate the expression of the IFN-λ receptor (*IFNLR1/IL10RB*) and to detect *in vivo* interferon-stimulated gene responses in individual immune cell populations. After filtering for high-quality cells, the authors included 263,668 cells, 146,408 cells from pegylated IFN-λ-treated, and 117,260 from placebo patients. ScRNAseq confirmed *in vivo* responses to pegylated IFN-λ in specific peripheral immune cells, but the treatment did not alter virus-specific adaptive immune responses. The antiviral effects of pegylated IFN-λ were observed despite a delayed T-cell response in older patients at risk of more severe outcomes (196).

Ryoo and collaborators conducted a comprehensive analysis to assess the effectiveness and safety of IFN (systemic or inhaled IFN-α, -β, and -λ) treatment in patients with COVID-19, stratified by the severity of their clinical condition. The meta-analysis incorporated data from 11 clinical trials encompassing 6,124 patients. The collective findings from this analysis indicated that, compared to a placebo, IFN therapy did not yield significant improvements in reducing mortality at day 28 or preventing the progression to mechanical ventilation in COVID-19 patients. However, it did exhibit a noteworthy increase in the rate of hospital discharge on day 14 when compared to the control group. In summary, IFN therapy was considered safe but did not exhibit favorable outcomes regarding critical clinical endpoints in COVID-19 patients, particularly those with more than moderate disease severity. Importantly, IFN therapy did not appear to worsen outcomes in patients with severe COVID-19. The study suggests that future clinical trials should focus on assessing the clinical efficacy of IFN therapy in patients with mild COVID-19 or at an earlier stage of the disease (27).

Research should investigate whether type IV IFN can be harnessed as a therapeutic protein. It would be significant to understand its potential therapeutic applications and evaluate its safety and efficacy in clinical settings (94). Addressing these questions and conducting further research will enhance our understanding of the type IV IFN system and its potential roles in immune response and therapy.

Taken together, the current evidence suggests that future clinical trials should focus on assessing the clinical efficacy of IFN therapy in patients with different clinical forms of COVID-19, from mild to severe and at different stages of the disease (27).

## 9 Conclusions

Timely coordinated cellular and humoral innate and acquired immune responses dictate the progression and severity of viral infections. One of the main effectors of antiviral innate immunity are the IFNs, which exert their antiviral activity through direct and indirect effects. IFNs interact with specific cell receptors and trigger activation signals for hundreds of genes that determine a broad spectrum of effects: inducing the antiviral state, inhibiting cellular proliferation, apoptosis activation, and stimulating the immune response. However, delayed IFN I synthesis could have opposite effects by stimulating the pro-inflammatory cytokine release and amplifying the pathologic process. The ability of SARS-CoV-2 proteins to interfere with IFN I response could explain the evolution and progression of infection in different individuals and indicate the potential therapeutic benefits of IFN in COVID-19.

Interferon treatments offer multiple benefits, including easy administration initiated by healthcare providers, potential acceleration of viral clearance, and quicker clinical improvement, especially when administered early in SARS-CoV-2 infection. Additionally, interferon therapy is associated with minimal reported side effects like temporary nausea and digestive problems.

However, the results of the available studies are contradictory, and to enable patients to benefit more effectively from IFN’s therapeutic use, there is a pressing need for standardization of interventional studies. Also, a deeper understanding of the timing and dynamics of IFN responses during SARS-CoV-2 infections is crucial for informing IFN-related therapies and vaccine development.

## Author contributions

GM: Writing – original draft, Writing – review & editing. MCC: Conceptualization, Writing – original draft, Writing – review & editing. RF: Writing – review & editing. CB: Writing – review & editing. LMD: Writing – review & editing. MC: Writing – review & editing, Figure design. R-EC: Writing – original draft, Writing – review & editing. RG: Writing – review & editing. SVB: Writing – review & editing. GB: Writing – review & editing. COV: Writing – original draft, Writing – review & editing.
